# Reducing Chemotherapy Waiting Times in the Ambulatory Setting of a Tertiary Cancer Centre Using a Design Thinking Approach

**DOI:** 10.3390/cancers15184625

**Published:** 2023-09-19

**Authors:** Wei-Ying Jen, Zhi Yao Chan, Yee Mei Lee, Noel Ng, Belinda Tan, Constance Teo, Yuet Peng Wong, Cheng Ean Chee, Yen-Lin Chee

**Affiliations:** 1 Department of Haematology-Oncology, National University Cancer Institute Singapore, Singapore 119074, Singapore; 2 Department of Pharmacy, National University Hospital, Singapore 119074, Singapore; 3 Division of Oncology Nursing, National University Cancer Institute Singapore, Singapore 119074, Singaporebelinda_tan@nuhs.edu.sg (B.T.); 4 Operations and Administration, National University Cancer Institute Singapore, Singapore 119074, Singapore

**Keywords:** design thinking, process redesign, chemotherapy wait times

## Abstract

**Simple Summary:**

Chemotherapy preparation and delivery is an enormously complex operation involving multiple teams. The complexity and safety requirements often impact on the timeliness of treatment. We aimed to take a human-centric approach and examine the processes required for chemotherapy provision. We identified key areas to redesign to improve the efficiency of care delivery. These included preparing chemotherapy in advance, optimizing scheduling and creating a data-driven system of continuous improvement.

**Abstract:**

Introduction: Chemotherapy is complex. We hypothesized that a design thinking approach could redesign preparatory processes and reduce wait times. Methods: A multidisciplinary process mapping exercise was undertaken to understand the current processes, followed by proposing and testing solutions. Proposals were selected based on desirability and feasibility. These focused on starting the morning treatments on time and scheduling pre-made regimens in these slots. The primary outcome measure was the time from the appointment to starting treatment. Treatments in the post-intervention study group were compared against a historical control group. Results: The median time to start morning treatment decreased by 46%, from 83 min (with an interquartile range 50–127) in the control group to 45 min (with an interquartile range of 24–81 min) in the study group (*p* < 0.001). This translated into an overall improvement for the day, with the median time to start treatment decreasing from 77 min (with an interquartile range of 40–120 min) to 47 min (with an interquartile range of 20–79 min) (*p* < 0.001). Pre-makes increased by 258%, from 908 (28.5%) to 2340 (71.7%) regimens (*p* < 0.001). The number of patients starting treatment within an hour of their appointment increased from 1688 (32.8%) to 3355 (62.3%, *p* < 0.001). Conclusion: We have shown that a data-driven, design thinking approach can improve waiting times. This can be adapted to improve other processes in an empathetic, sustainable manner.

## 1. Introduction

Chemotherapy is a critical component of cancer treatment. Over 80% of healthcare encounters for chemotherapy occur in the ambulatory setting [1], as most chemotherapy is delivered parenterally. Long waiting times for ambulatory chemotherapy are common, and adversely affect patient perceptions of the quality of delivered care [2], as well as threatening overall capacity at the treatment center [3].

One of the key drivers of waiting time is the complexity and dynamic nature of chemotherapy delivery [4]. It involves cross-functional healthcare teams and comprises dosing, scheduling, safety checks, drug compounding and administration [5,6]. Patient factors, such as adherence and fitness for treatment, together with rising drug costs [7,8,9], also influence the capability of a treatment center to introduce measures aimed at reducing waiting times. Coupled with team silos [10], legacy systems that are unable to integrate with each other, workflows that have to be adapted to those systems instead of prioritizing care provision [11], and escalating workload, efficient chemotherapy delivery is increasingly challenging, resulting in negative staff and patient experience. Tackling these issues requires a deep understanding of the entire process from multiple perspectives and innovative, data-driven approaches, possibly with the utilization of artificial intelligence [12].

Design thinking is a creative, person-centered, solution-based approach suited to tackle complex problems in healthcare [13]. It comprises five steps: (a) empathize and understand the user’s needs; (b) define the user’s needs and problems; (c) ideate; (d) prototype ideas into initial solutions; and (e) test the prototype and collect feedback to iterate the process. Given the complexity of the chemotherapy preparation process and the multiple touchpoints from varying staff members and patients, we sought to use this approach. We hypothesized that a multidisciplinary team using a data-driven, design thinking approach to restructure chemotherapy workflows could improve staff user experience, reduce time to treatment and improve operational efficiency at our chemotherapy center. 

## 2. Methods

This was a prospective single-arm study with a historical control group conducted in the ambulatory setting. We included consecutive outpatients treated from 1 November 2019 to 31 October 2020. Patients were split into two groups: a historical control group (1 November 2019 to 30 April 2020) and a post-intervention study group (1 May to 31 October 2020). 

We conducted the study in two phases, using the five-step design thinking process. Phase 1 comprised the empathize, define, and ideate steps, while Phase 2 comprised the prototyping and testing steps. Fortnightly meetings were held to review outcomes and to iterate and refine the approaches.

### 2.1. Phase 1: Empathise, Define, and Ideate

We first observed and engaged with the users to understand their needs and objectives. Members of all healthcare teams involved in the chemotherapy process were invited to participate in the exercise. Over 50 staff members, including doctors, nurses, pharmacists, patient service associates and administrative staff, took part in the week-long exercise, culminating in an ideation workshop. Staff members shadowed both patients and staff from different job groups to facilitate mutual understanding and empathy. Staff were encouraged to make their own notes on the processes observed. They were provided with a shadowing template, which urged them to note down the computer systems involved, time taken for tasks, pain points, emotional states, and suggestions for improvement from the person they were shadowing. During this period, an anonymous survey was sent to all staff, asking them to identify areas of redundancy or frustration in their work processes, and suggest how their workday may be improved.

After the shadowing exercise, feedback was collated and a value stream map of the chemotherapy process and patient journey with relevant pain points was created. We synthesized these findings to define the user problems as clear problem statements. In the ideation phase, a diverse group of participants worked together to brainstorm ‘how might we’ statements to address the pain points. The participants reviewed the ‘how might we’ statements, settling on those with the best balance between desirability and feasibility to take forward to Phase 2 for prototyping and testing. 

The main problem statement was: “60% of patients are waiting more than an hour from their appointment time to start treatment.” The following “how might we” questions were used for the ideation phase: 

1. How might we decrease chemotherapy waiting time?

2. How might we ensure timely chemotherapy prescriptions?

3. How might we increase advanced chemotherapy preparations (pre-makes) for patients?

4. How might we ensure that only regimens which can be pre-made are listed in the morning? Morning slots were defined as appointments between 0830 and 1030.

5. How might we track our progress and identify evolving issues?

6. How might we improve inter-team communication?

### 2.2. Phase 2: Prototype and Test

In the prototype phase, we discussed different proposals for different user problems before the solutions were put into motion in the test phase. During the ideation phase, staff were divided into six multi-disciplinary groups and generated answers to the “how might we” questions. The entire group then discussed these answers, plotting the suggestions on a graph with desirability on the y-axis and feasibility on the x-axis. Interventions that were both desirable and feasible were chosen for testing. Examples of suggestions, which were not feasible, included having chemotherapy orders and patient lab results completed and available two days in advance of the chemotherapy order (pre-treatment parameters may not have recovered by this time), and having real-time chair availability and chemotherapy status available in the infusion center with a real-time dashboard (due to lacking technology at our center at the time). Solutions were introduced simultaneously, with continuous refinement. 

We concluded that starting the morning on time and increasing the proportion of chemotherapy prepared in advance, known as pre-makes, would exert the biggest influence on chemotherapy waiting times. This would reduce the number of morning infusions overrunning into later slots and allow the pharmacy to use the morning to pre-make short-expiry regimens for the afternoon. New workflows were drafted, iterated, and implemented on 1 May 2020 with the following major changes:

1. Blood tests and physician consults had to be carried out at least a day in advance of the chemotherapy appointment, with physicians reminded to complete chemotherapy orders by 3 p.m. the day before. This allowed time for pharmacists to review the chemotherapy order and perform safety checks in advance. For regimens eligible to be pre-made, this also allowed the drugs to be made up the day before the infusion appointment. 

2. Pharmacy teams were re-organized, with dedicated teams for inpatient chemotherapy, same-day ambulatory chemotherapy, pre-makes and dispensing. 

3. Prior to the workshop, the scheduling team mainly comprised nursing staff, given the complexity of chemotherapy scheduling. Following the workshop, nurses were re-deployed to enhance nursing manpower, while a dedicated group of administrative staff took over scheduling. To aid the new team, all chemotherapy regimens were consolidated into a detailed spreadsheet containing properties such as total infusion duration, pre-make eligibility and other scheduling characteristics, such as the number of days per cycle and day restrictions (for example, 48 h infusional chemotherapy cannot start on a Friday as the infusion center is not open on a Sunday, precluding pump removals). Pre-make eligibility was determined by a team of pharmacists based on regimen cost and stability. Other scheduling characteristics included whether labs were required the day before treatment (e.g., prior to day 8 of a regimen dosed on days 1, 8 and 15), chair restrictions (e.g., cohorting for infection control), infusion duration for subsequent cycles (e.g., the first dose of rituximab is run over 6–8 h, while subsequent infusions take 90 min if there are no reactions the first time), and regimen synonyms. Regimen synonyms were provided by the entire team and were used to ensure that searches returned results of interest to the user. Scheduling rules were also accorded a priority such that pre-makes were always prioritized for early morning chemotherapy slots, and long infusions should not start after a time that would result in the infusion finishing after the infusion center’s closing hours. This was made searchable via an Excel^®^ formula (Microsoft, Redmond, WA, USA), which matched the search terms against all the regimens indexed in the reference spreadsheet, returning all the characteristics (regimen booking duration, chair and day restrictions, etc.) the schedulers needed to book chemotherapy slots correctly. Pre-makes and drugs that did not require compounding, such as pre-filled syringes, were prioritized for morning appointment slots. 

4. An anonymized database was designed to track chemotherapy delivery and care provision outcomes. An Excel^®^ algorithm (Microsoft, Redmond, WA, USA) was written to link data extracted from appointment, queue management and chemotherapy systems. Outcome targets were agreed on and tracked daily. These were made accessible to all staff via an online dashboard. 

5. The workgroup met weekly to discuss targets, barriers and iterate workflows. Daily, intra-group communication was facilitated by TigerConnect^®^ (TigerConnect, Santa Monica, CA, USA).

### 2.3. Staff Survey

Three months after implementing our changes, we created an anonymous survey to assess the impact of the changes on staff satisfaction. Staff were asked a mixture of nine qualitative and quantitative questions, two of which were open-ended questions identical to the pre-implementation survey. The full list of questions is provided in the Supplementary Appendix (online only). The three main quantitative questions were scored on a five-point Likert scale (strongly agree, agree, neutral, disagree and strongly disagree) and were worded as follows:

1. It is easier to do my job now compared to the same period last year.

2. I am more satisfied with my job compared to the same period last year.

3. The patients I meet are more satisfied with their chemo delivery compared to the same period last year.

As a similar survey had not been carried out pre-intervention, we worded the questions to assess for improvement in the domains assessed. Answers to all questions were mandatory.

### 2.4. Analysis

Data were extracted from our institution’s appointment, queue management and chemotherapy ordering systems. The queue management system tracks when a patient arrives and interacts with different touchpoints along their journey through the Cancer Centre. Patients were defined as being on time if they arrived 30 min or more before their appointment time. They are asked to do so because the pre-treatment processes such as screening, drug dispensing and obtaining venous access take approximately 30 min. The chemotherapy ordering system in our institution is a custom-built Hypertext Markup Language (HTML)-based system with prescribing, pharmacy and administration modules. It stores all data related to chemotherapy, including regimen details, prescription time, intended treatment date, date changes, order review times, drug compounding times, safety checks and administration times of each drug. 

The primary outcome measure was the difference between appointment time and treatment start time, in minutes. Secondary outcome measures included (a) the proportion of pre-made chemotherapy; (b) the number of patients starting treatment within an hour of the appointment time; and (c) the number of patients finishing treatment after 6 p.m. 

Continuous data are reported as median (25th–75th percentile) and were analyzed with the Mann–Whitney U test, while categorical data were assessed with the chi-square test. The analysis was performed using SPSS v22 (IBM, Armonk, NY, USA).

## 3. Results

The value stream map detailing the patient and chemotherapy journey is shown in Figure 1 of the Supplementary Appendix (online only). In brief, the pain points identified pertained to difficulties encountered by different job groups during the chemotherapy preparation process. The longest delay identified was the time required to order, review and prepare the chemotherapy for patients having a physician consult on the same day as their chemotherapy treatment (red bars in Supplemental Figure S1). 

Results are summarized in Table 1. From 1 November 2019 to 31 October 2020, 24,733 treatments were completed at the Cancer Centre. Of these, 10,487 (42.4%) were in the 0830 to 1030 slots prioritized for pre-made chemotherapy. 

**Figure 1:**
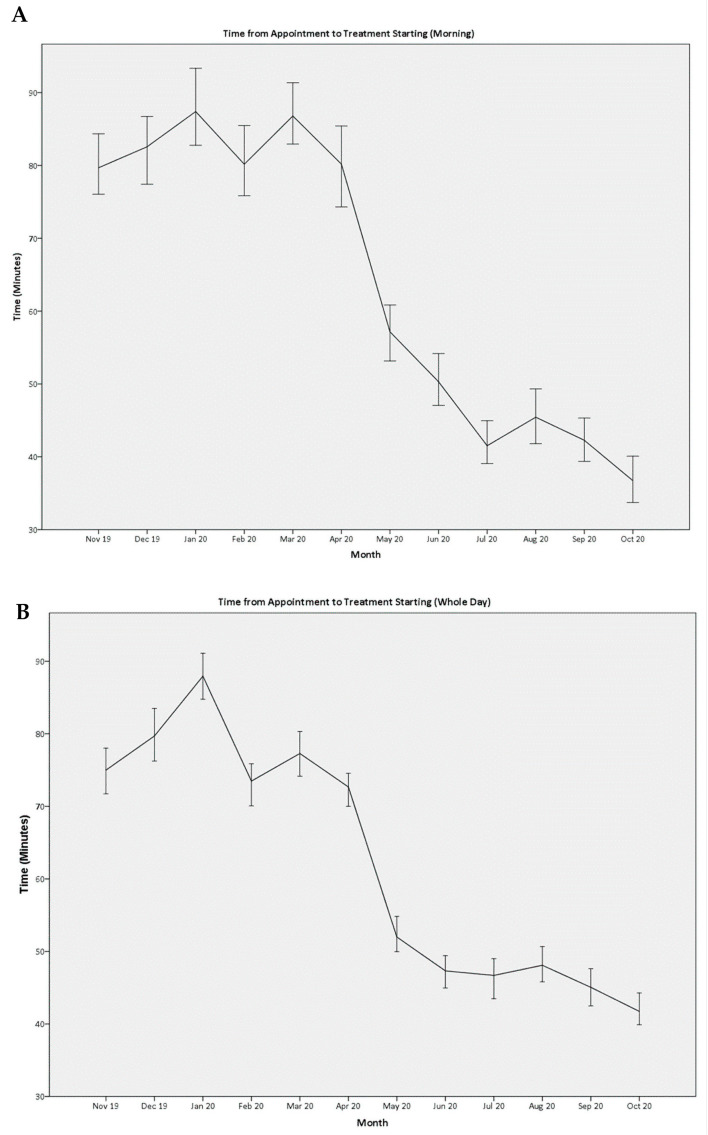
Median time to start treatment for patients listed in the morning (0830–1030) slots (**A**) and for the whole day (**B**). Measures were implemented at the end of April 2020. There is a clear decrease in waiting time after 1 May 2020. Figure 1B shows that the waiting time also decreased for the whole day, demonstrating that focusing on the morning slots did not have an adverse effect on patients in the later part of the day. Rather, these patients also benefited from the earlier patients completing treatment in a timelier fashion.

For patients with appointments in the morning, the median time to start treatment decreased from 83 (50–127) minutes in the control group to 45 (24–81) minutes in the study group (*p* < 0.001). The sustained and continued improvement in month-on-month median waiting times are shown in Figure 1A. This translated into an overall improvement for the day, with the median time to start treatment decreasing from 77 (40–120) minutes to 47 (20–79) minutes (*p* < 0.001, Figure 1B).

**Figure 2:**
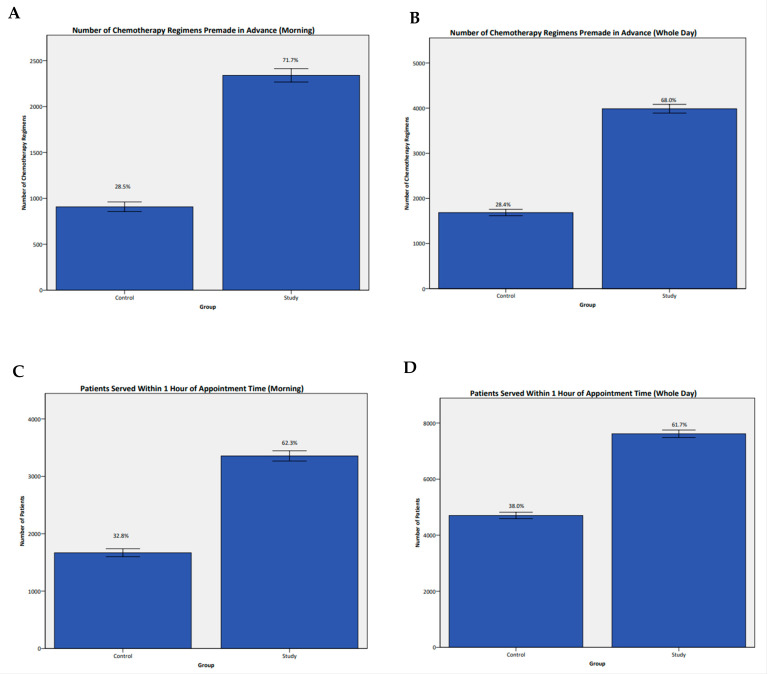
Number of chemotherapy regimens pre-made in advance for patients in the morning (**A**) and for the whole day (**B**). Graphs (**C**) and (**D**) show the number of patients starting treatment within one hour of their appointment time for morning slots and the whole day, respectively.

There were 5936 (47.9%) regimens eligible for pre-make in the control group, compared to 5869 (47.5%) in the study group (*p* = 0.344). Of the regimens eligible to be pre-made, the proportion of regimens prepared a day in advance of the chemotherapy appointment increased from 1685 (28.4%) in the control group to 3989 (68.0%) in the study group (*p* < 0.001, Figure 2B).

For patients in morning slots, there were 3189 (61.9%) regimens eligible for pre-make in the control group, compared to 3264 (61.1%) in the study group (*p* = 0.117). Of the regimens eligible to be pre-made, the proportion of regimens prepared a day in advance of the chemotherapy appointment increased from 908 (28.5%) in the control group to 2340 (71.7%) in the study group (*p* < 0.001, Figure 2A).

To assess the accuracy of the scheduling team, regimens eligible for pre-make and pre-filled syringes were considered together. Of the 11,072 regimens scheduled in morning slots, 5402 (48.9%) were in the control group, and 5670 (51.2%) were in the study group. In the control group, 4005 (74.1%) regimens were correctly scheduled, compared to 4505 (79.5%) regimens in the study group (*p* < 0.001).

The number of patients with morning appointments starting treatment within 1 h of their appointment time increased from 1688 (32.8%) in the control group to 3355 (62.3%) in the study group (*p* < 0.001, Figure 2C). For the entire day, this increased from 4702 (38.0%) in the control group to 7617 (61.7%) in the study group (*p* < 0.001, Figure 2D). This resulted in fewer patients finishing treatment after 6 p.m.—2517 (20.3%) in the control group versus 1209 (9.8%) patients in the study group (*p* < 0.001).

A total of 3823 (36.5%) patients with morning appointments arrived more than 30 min prior to their appointment time, while 2634 (25.1%) of patients arrived after their appointment time. Patients who arrived on time experienced the shortest time to treatment, with a median of 61.5 (30.5–100.5) minutes in the control group and 24.5 (6.2–49.1) minutes in the study group (*p* < 0.001). In contrast, patients who arrived after their appointment time experienced longer waiting times, with a median time to treatment of 119.1 (85.4–173.2) minutes in the control group and 83.3 (54.9–148.1) minutes in the study group (*p* < 0.001).

92 staff members responded to the anonymous post-intervention survey, resulting in a response rate of 93.9%. Among the staff members who responded, 51 (55.4%) were nurses, 29 (31.5%) were pharmacists or pharmacy technicians, 10 (10.9%) were patient service associates and two (2.2%) were operations staff. 40 (43.5%) respondents agreed that it was easier to do their job compared to the same period the previous year, with 43 (46.7%) responding neutrally. 33 (35.8%) of respondents were more satisfied with their jobs compared to the previous year, with 46 (50.0%) responding neutrally. 47 (51.1%) believed patients were more satisfied with chemotherapy delivery, with 38 (44.6%) responding neutrally. 

## 4. Discussion

In this study, we show that a multi-disciplinary working group, using a human-centered, empathetic, and collaborative approach through design thinking, can reduce waiting times for chemotherapy in the ambulatory setting. These measures increased staff job satisfaction and perceptions of care delivery. The project was undertaken with no added expenditure, during a raging pandemic, which resulted in manpower constraints due to team segregation [14] and new workflows to protect patients and staff.

The design thinking methodology starts with developing an empathic understanding of user needs to develop effective solutions. This involves reframing problems in person-centered way, with collective ideation and continuous refinement [15]. This solution-based approach focuses on ‘how?’ questions and differs from conventional problem-based approaches, which ask ‘why?’ and ‘what?’. The concept of design thinking is relatively new to healthcare but can be integrated with traditional quality improvement tools to effect change. In essence, design thinking focuses on the understanding people, while quality improvement tools seek to understand processes [16]. We suggest that both are fundamentally necessary to effect meaningful change, especially in healthcare settings defined by empathetic care delivery.

The application of this methodology comprehensively identified and addressed aspects of the chemotherapy scheduling, ordering, preparation, and administration processes that were leading to delays. These issues have also been identified in other studies [3,17,18,19,20,21]. Our approach is unique in that it holistically incorporates proposals to address these issues by staff with front-line knowledge of the problems, and with the ability to assess the feasibility of proposed solutions. In addition, changes to key areas in concert helped to ensure that progress was made overall, rather than stymied when one problem area was addressed with another left hanging. 

Chemotherapy is a high-alert medication, demanding rigorous checks and verifications [22,23,24]. Errors can lead to serious adverse events. Our strategy of requiring chemotherapy to be prescribed a day in advance allows pharmacists the time they need to verify and check chemotherapy orders, ensuring safety while also prioritizing efficient drug delivery at the time of the appointment. The focus on preparing chemotherapy in advance and prioritizing morning slots for patients with such regimens also gives the pharmacists more breathing room on the day of chemotherapy. A large proportion of the regimens administered at our center cannot be pre-made because of cost and stability concerns. With morning treatments already pre-made, pharmacists are free to use the time to review and compound these high-cost drugs, limiting wastage while maintaining efficiency.

We also showed that, even though chemotherapy scheduling is complex [21], staff unfamiliar with regimens and their administration can accurately perform scheduling when information is consolidated and presented accessibly. Prior to our intervention, scheduling was being done by nurses due to the domain knowledge required. The indexing of regimen information and presentation via a search algorithm allowed nursing manpower to be redeployed back to chemotherapy administration, enhancing a valuable resource with no added cost. The efficacy of the algorithm was demonstrated by the increase in scheduling accuracy, despite administrative staff with no domain knowledge taking over the process.

The successful implementation of approaches such as the one we describe here can be of benefit to healthcare managers and society as a whole. For healthcare managers, this approach not only enables the optimization of work processes, improving operational efficiency but also enhances patient satisfaction by reducing chemotherapy waiting times, a critical factor in patient care [25]. The fact that these improvements were achieved without any increase in budget and by using accessible software makes it a cost-effective solution, which is particularly important in a healthcare landscape that is constantly under pressure to deliver more with less [26]. Additionally, enhanced operational efficiency can free up resources, both human and financial, which can then be redirected to other critical areas of healthcare, benefiting the broader community [27].

This study has several limitations. Firstly, there was no cost data available in terms of cost to the healthcare system or cost to the patient. Nevertheless, all the measures we implemented were designed by members of the team using readily available software already pre-installed on hospital computers. No additional expenditure for solution design, hardware or software was necessary because of this project. We plan to analyze the financial impact of the interventions in the future. We also do not have data on whether there were medium or long-term adverse effects of our intervention. We plan to conduct a retrospective study once the interventions have been established to determine if there have been any unexpected adverse effects because of our interventions.

Secondly, although we showed that chemotherapy waiting times can be dramatically reduced through process change, we do not have data to show how such improvements impacted patients’ perceptions and experience of their treatment. Regardless, it has previously been shown that patient satisfaction scores, adherence and perception of treatment provision correlate with waiting times [28,29,30]. In addition, the majority of staff surveyed felt that patients were more satisfied with chemotherapy delivery compared to the preceding year. We believe this is a good indicator for patient satisfaction, since the majority of respondents were front-line, patient-facing staff.

Thirdly, we found that patient punctuality was a major factor in starting chemotherapy on time. Late-coming adversely affected treatment start times for both the late patient and the subsequent patient scheduled after. We showed that patients who were consistently on time started treatment earlier, even in the pre-intervention control group. Although fewer patients were on time in the study group, there was still a significant improvement in time to treatment for patients who were on time, late and overall. It is possible that patients were later in the study group because of delays due to temperature screening upon arrival at the hospital. Targeting patient punctuality could further improve time to treatment and create more capacity.

Lastly, we were unable to include a contemporary control group due to the complexity of the chemotherapy delivery process. This meant that we were unable to implement the changes for only a subset of the patients being treated at the center, as it would have been impossible to identify them with the technology that we had available at the time, in addition to the restrictions imposed by the ongoing COVID-19 pandemic. The choice of a historical control is a pragmatic one; we chose a group treated immediately before the changes to minimize the impact of other unknown changes on our outcomes.

## 5. Conclusions

We have shown that a multidisciplinary group using a data-driven, design thinking approach to address team silos, reorganize and track work processes can effectively bring about change in outpatient chemotherapy delivery processes. These changes were well-received by staff members and resulted in a significant 46% reduction in chemotherapy waiting times for patients. Changes were made with no additions to our budget and by using accessible software. We plan to analyze the effect of these changes on cost savings in terms of capacity creation and overtime claims for staff in a future study. Such an approach can be adapted to improve workflow processes in other areas of healthcare in an empathetic, sustainable manner.

## Figures and Tables

**Table 1:** Outcome measures for the study and control groups. IQR, interquartile range.

Outcome Measure		Control Group	Study Group	*p* Value
**Morning (between 0830 and 1030 only)**	Number of orders	5402	5670	
	Number of treatments completed	5149	5338	
	Arrived 30 min or more before appointment time, *n* (%)	2032 (39.4)	1791 (33.5)	<0.001
	Arrived after appointment time, *n* (%)	1330 (24.9)	1304 (25.3)	0.106
	Premade chemotherapy, *n* (%)	908 (28.5)	2340 (71.1)	<0.001
	Median time to start treatment, minutes (IQR)	83 (50–127)	45 (24–81)	<0.001
	Patients starting treatment within 1hr, *n* (%)	1688 (32.8)	3355 (62.3)	<0.001
**Whole Day**	Number of orders	13,154	13,175	
	Number of treatments completed	12,380	12,353	
	Arrived 30 min or more before appointment time, *n* (%)	6697 (54.1)	5526 (44.7)	<0.001
	Arrived after appointment time, *n* (%)	2228 (18.0)	2266 (18.3)	0.414
	Premade chemotherapy, *n* (%)	1685 (28.4)	3989 (68.0)	<0.001
	Median time to start treatment, minutes (IQR)	77 (40–120)	47 (20–79)	<0.001
	Patients starting treatment within 1 h, *n* (%)	4702 (38.0)	7617 (61.7)	<0.001
	Patients finishing after 6 pm, *n* (%)	2517 (20.3)	1209 (9.8)	<0.001

## Data Availability

Raw, unprocessed data are unavailable due to privacy restrictions.

## Supplementary Materials

The following supporting information can be downloaded at: https://www.mdpi.com/article/10.3390/cancers15184625/s1, Figure S1: Value Stream Map of Patient Journey and Chemotherapy Preparation Process and Pain Points.
