# Supplementary material for: Reducing Chemotherapy Waiting Times in the Ambulatory Setting of a Tertiary Cancer Centre Using a Design Thinking Approach
Source: Cancers (Basel). 2023 Sep 19;15(18):4625. doi: 10.3390/cancers15184625 (PMC10526492; doi:10.3390/cancers15184625)
Supplement: Supplementary file 1 [file cancers-15-04625-s001.zip › cancers-2592775-supplementary.pdf]

Supplementary Appendix

Post-Intervention Survey Questions

1. I am a…
    a. Nurse
    b. Pharmacist
    c. Pharmacy Technician
    d. Patient Service Associate
    e. Operations Staff
2. It is easier to do my job now compared to the same period last year.
    a. Strongly agree
    b. Agree
    c. Neutral
    d. Disagree
    e. Strongly disagree
3. I am more satisfied with my job compared to the same period last year.
    a. Strongly agree
    b. Agree
    c. Neutral
    d. Disagree
    e. Strongly disagree
4. The patients I meet are more satisfied with their chemotherapy delivery compared to the same period last year.
    a. Strongly agree
    b. Agree
    c. Neutral
    d. Disagree
    e. Strongly disagree
5. What changes have made your work day easier or more efficient? Select one or more options.
    a. Starting registration earlier (7.45 am)
    b. Not having to rely on EQMS to call patients in the morning
    c. No blood tests on the same day as chemotherapy
    d. No chemotherapy on the same day as physician consult
    e. More chemotherapy ordered the day before
    f. More chemotherapy pre-made the day before
    g. Pre-printed premedication stickers
    h. More manpower
    i. A forum to give feedback about workflows and continuous improvement
    j. Having objective, data-driven targets to aim for daily
    k. Rounding for medication dispensing (e.g. morphine syrup_
    l. Electronic home delivery workflow
    m. None of the above
    n. Other (please specify): ________
6. Which of these changes are not sustainable? Select one or more options.
    a. Starting registration earlier (7.45 am)
    b. Not having to rely on EQMS to call patients in the morning
    c. No blood tests on the same day as chemotherapy

d. No chemotherapy on the same day as physician consult
e. More chemotherapy ordered the day before
f. More chemotherapy pre-made the day before
g. Pre-printed premedication stickers
h. More manpower
i. A forum to give feedback about workflows and continuous improvement
j. Having objective, data-driven targets to aim for daily
k. Rounding for medication dispensing (e.g. morphine syrup_
l. Electronic home delivery workflow
m. None of the above
n. Other (please specify): ________

7. If you feel the changes are not sustainable, please elaborate why here and suggest alternatives.
8. What are some of the challenges which make it difficult to do your job?
9. What would you change to make your work day more efficient?

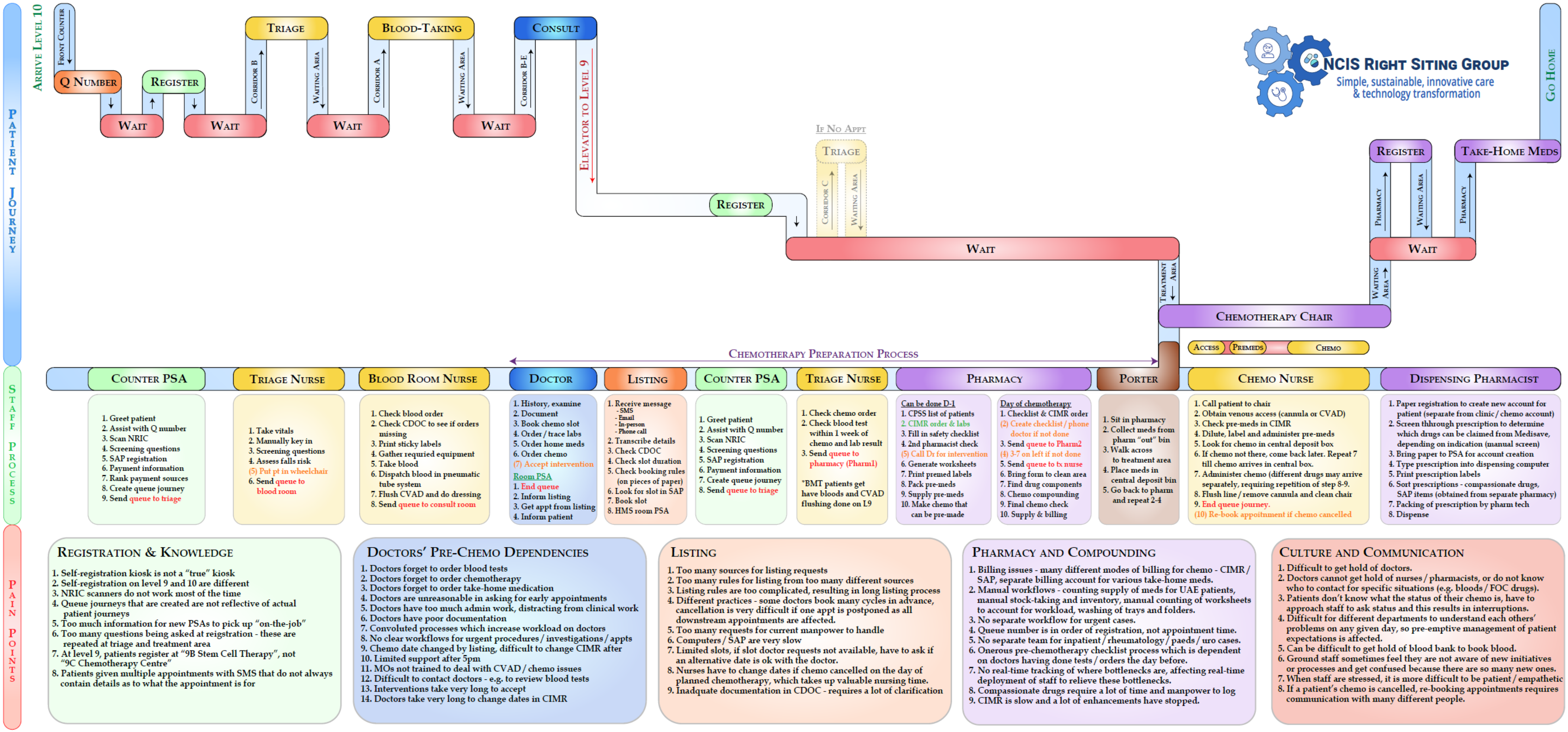

Figure S1. Value Stream Map of Patient Journey and Chemotherapy Preparation Process and Pain Points.
